# Problematic Social Media Use Among University Entrance Test‐Takers: Prevalence, Psychosocial Factors, and a Mediation‐Moderation Model

**DOI:** 10.1002/brb3.70911

**Published:** 2025-09-25

**Authors:** Mohammad Majharul Islam, Shikder Mohammad Rakibul Hasan, Akihiro Masuyama, Farzana Tayeeba, Md. Faruk Islam, Nayeem Hasan Obhi, Miftahul Jannat Tahia, Ayesha Siddika Khan Sayma, Moneerah Mohammad ALmerab, Debasruti Ghosh, Saurabh Raj, Mohammed A. Mamun, Firoj Al‐Mamun

**Affiliations:** ^1^ CHINTA Research Bangladesh Dhaka Bangladesh; ^2^ Centre For Qualitative Research Dhaka Bangladesh; ^3^ Department of Psychology University of Dhaka Dhaka Bangladesh; ^4^ Department of Psychology Aichi University of Education Kariya Aichi Japan; ^5^ Department of Government and Politics Jahangirnagar University Savar, Dhaka Bangladesh; ^6^ Department of Public Health and Informatics Jahangirnagar University Savar, Dhaka Bangladesh; ^7^ Department of Pharmacy Jahangirnagar University Savar, Dhaka Bangladesh; ^8^ Department of Psychology, College of Education and Human Development Princess Nourah Bint Abdulrahman University Riyadh Saudi Arabia; ^9^ Department of Psychology, MDDM College Babasaheb Bhimrao Ambedkar Bihar University Muzaffarpur Bihar India; ^10^ Department of Psychology, Ramdayalu Singh College Babasaheb Bhimrao Ambedkar Bihar University Muzaffarpur Bihar India; ^11^ Department of Public Health University of South Asia Dhaka Bangladesh

**Keywords:** anxiety, depression, perceived social support, problematic social media use, psychological distress, stress

## Abstract

**Background:**

Social media has become integral to daily life, but problematic social media use (PSMU) is an emerging public health concern. Few studies have specifically examined PSMU among university admission test‐takers. This study aimed to investigate the prevalence and predictors of PSMU, the mediating role of social media use duration and the moderating effect of perceived social support on the relationship between psychological distress and PSMU among university entrance test‐takers in Bangladesh.

**Method:**

A cross‐sectional study was conducted in February 2025, involving 1139 students preparing for university admission tests. Data on sociodemographic, admission‐related factors, mental health symptoms, perceived stress, social support, and PSMU were collected. Data analysis involved Chi‐square tests, logistic regression, and structural equation modeling (SEM) using IBM SPSS 26 and R (lavaan package).

**Results:**

The prevalence of PSMU was 21.2%. Logistic regression analysis revealed that social media use duration, cigarette smoking, fracture in body parts, depression (OR = 1.60, 95% CI = 1.10–2.34), and high stress (OR = 1.65, 95% CI = 1.03–2.64) had significantly increased odds of developing PSMU. Participants with moderate social support had higher likelihood of PSMU (OR = 1.51, 95% CI: 1.05–2.16). SEM analysis indicated that anxiety (*β* = 0.37, *p* = 0.009) and stress (*β* = 0.27, *p* < 0.001) had significant direct effects on PSMU, whereas depression did not directly influence PSMU. Social media use duration significantly mediated 24.7% of the effect of stress on PSMU (indirect *β* = 0.089, *p* = 0.003), but no significant mediation was found for anxiety or depression. Perceived social support did not significantly moderate the relationships between psychological distress and PSMU.

**Conclusion:**

Anxiety, stress, and social media usage duration contribute to PSMU. These results inform targeted interventions to mitigate PSMU behaviors and support mental health in this vulnerable group.

## Graphical abstract

Problematic social media use (PSMU) affected 21.2% of Bangladeshi university entrance test‐takers. Smoking, fractures, stress, depression, moderate social support, and longer social media use duration were key risk factors. Social media use duration mediated the effect of stress on PSMU, while perceived social support showed no moderation.

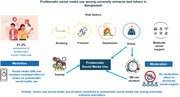

## 1 Introduction

In the digital age, social media has become an integral part of daily life, transforming the ways in which user communicate, access information, and build virtual communities. Social media refers to third‐party internet‐based platforms that enable user interaction and content sharing across digital networks. Widely used platforms such as Facebook, WhatsApp, Instagram, Twitter, and TikTok, connect billions of users globally, transcending geographic and cultural boundaries. With rapid urbanization and digitalization, social media use has expanded significantly both in Bangladesh and worldwide (DataReportal 2021; Kemp 2025). According to the Digital 2021 report, global social media usage has increased by 1.5 times over the past five years (DataReportal 2021). A more recent projection suggests that by 2025, around 64% of the global population, approximately 5.24 billion individuals, will be social media users (Kemp 2025).

Social media offers several benefits, including enhanced access to information (Drahošová and Balco 2017), increased social support, opportunities for self‐expression and creativity, and enhanced media literacy (Naslund et al. 2020). However, growing evidence suggests that excessive or compulsive use, often termed “social media addiction,” can have adverse effects (Naslund et al. 2020). Among adolescents and young adults, problematic use has been linked to disrupted sleep, life satisfaction, reduced academic performance, and elevated risks of psychiatric disorders (Woods and Scott 2016; Masoed et al. 2021; Sümen and Evgin 2021). Studies from developing countries further highlight associations between problematic social media use (PSMU) and stress, loneliness, low self‐esteem, and anxiety across various age groups (Glazzard and Stones 2020; Hou et al. 2019). Additional findings have linked PSMU to feelings of loneliness and isolation, cyberbullying, depression, online harassment, and suicidality (Glazzard and Stones 2020; Naslund et al. 2020).

International evidence has highlighted the public health implications of PSMU. A recent meta‐analysis of 53 studies involving approximately 59,928 participants found a strong correlation between social anxiety and PSMU, moderated by gender, platform type, and publication year (Wu et al. 2024). Approximately 18.4% of university students exhibited symptoms of social media addiction globally (Salari et al. 2025), while a meta‐analysis across 32 countries estimated the pooled prevalence of social media addiction at 24% (Cheng et al. 2021). In China, PSMU among college students was predicted by female gender, impulsivity, anxiety, social anxiety, and negative attentional bias (Zhao et al. 2022). These usage patterns have been consistently associated with poor sleep, low self‐esteem (Woods and Scott 2016; Köse and Doğan 2019), and a range of psychiatric and behavioral conditions, including poor self‐regulation, fear of missing out (FOMO), negative affectivity, and social comparison orientation (Blease 2015; Servidio et al. 2024). Additionally, PSMU has been linked to anxiety and depression in a Scottish study (Woods and Scott 2016), while a Turkish study showed a significant association between perceived stress levels and PSMU (Sarialioğlu and Oluç 2024). These findings highlight the importance of exploring depression, anxiety, and stress as predictors of PSMU in adolescent and youth populations.

The Compensatory Internet Use Theory (CIUT) provides a theoretical framework for understanding these associations. It posits that individuals may engage in online behaviors to escape from negative emotional states or compensate for real‐life challenges, such as stress, loneliness, or dissatisfaction (Kardefelt‐Winther 2014). This aligns with the self‐medication hypothesis, which suggests that those experiencing psychological distress may use digital technologies to regulate mood (Shiraly et al. 2024). Although such use may provide temporary relief, it often becomes maladaptive over time, contributing to compulsive patterns of use and deteriorating mental health (Sümen and Evgin 2021).

One behavioral mechanism linking psychological distress to PSMU is social media use duration, which may serve as a mediator in this relationship. Individuals reporting higher levels of depression, anxiety, or stress are more likely to spend extended time on social media (Francisquini et al. 2025), and prolonged use is strongly associated with PSMU (Al‐Mamun et al. 2022; Shanshal et al. 2024). Despite this evidence, the mediating role of social media use duration remains under‐investigated, particularly among high‐stress populations such as university entrance exam candidates, who may be especially vulnerable to excessive use as a stress‐regulation strategy.

In contrast, perceived social support may act as a moderator that buffers the relationship between psychological distress and PSMU. Support from family, friends, or significant other person's is known to reduce vulnerability to emotional and behavioral problems (Zimet et al. 1988). According to the stress‐buffering hypothesis, individuals with greater perceived support are less likely to rely on maladaptive coping strategies such as excessive social media use. Empirical evidence shows that perceived social support is inversely associated with PSMU and positively associated with mental health outcomes (Lin et al. 2021; Zhao et al. 2021). For instance, a previous study reported that individuals with greater support reported less PSMU and greater mental well‐being (Lin et al. 2021), while another study reported that those receiving higher levels of support from family and friends reported significantly lower levels of social media overuse (Bilgin and Taş 2018). These findings suggest that perceived social support may attenuate the strength of the relationship between psychological symptoms and PSMU.

University admission test‐takers in Bangladesh are especially vulnerable to PSMU due to a unique confluence of psychosocial stressors. Typically aged 18–24 years, these students face immense academic competition, uncertain career prospects, and high societal expectations, all of which amplify their risk for maladaptive social media engagement (Al‐Mamun et al. 2022). Admission to public universities in Bangladesh is intensively competitive, for instance, in the 2024–2025 undergraduate admission cycle at the University of Dhaka, only 5.93% of candidates passed the “Ka” (Science) unit exam, highlighting the scale of competition (Wasif 2025). Many students prepare for one to two years, often in isolation, enduring chronic stress, sleep deprivation, and heightened anxiety (Al‐Mamun et al. 2024). As a coping mechanism, these students may increasingly turn to social media, aligning with the CIUT's proposition that digital engagement may serve to alleviate emotional distress.

Although empirical studies on PSMU in Bangladesh have expanded, research specifically targeting admission test‐taking students remains notably absent. Prior studies have focused on high school, university, or medical college students, reporting problematic Facebook use of 29.4% among medical college students (Karim et al. 2023) and 29.1% among university students (Al‐Mamun et al. 2022). PSMU has been linked to anxiety, depression, stress, and lifestyle factors, including poor sleep, irregular physical activity, and certain personality traits (Ahmed et al. 2022; Al‐Mamun et al. 2022; Islam et al. 2021; Karim et al. 2023).

Importantly, a recent study estimated the prevalence of digital addiction at 33.1% among university entrance test‐takers, identifying student status, satisfaction with mock test performance, monthly expenditure during admission test preparation, and depression as significant factors (Al‐Mamun, Hasan, et al. 2024). However, this study did not specifically investigate PSMU or model its psychological and behavioral pathways, leaving a critical gap in understanding. To the best of our knowledge, no prior studies either in Bangladesh or globally have specifically investigated PSMU among university admission test‐takers, despite their heightened vulnerability. This group experiences extreme academic pressure, career uncertainty, societal and familial expectations, and emotional instability, making them highly susceptible to maladaptive coping strategies, such as compulsive social media use.

Unlike regular enrolled university students, admission test‐takers often prepare in social isolation, frequently attend long and demanding coaching sessions, and face repeated examination cycles. These conditions can foster emotional disconnection and increase reliance on digital media for emotional relief. Combined with psychological distress, excessive screen time may contribute substantially to the development of PSMU in this high‐risk population.

Given the lack of empirical research, the present study aims to explore the psychological and behavioral mechanisms underlying PSMU among Bangladeshi university admission test‐takers. Specifically, the study seeks to identify key factors associated with PSMU in this population and to elucidate the complex relationships between psychological distress, social media use, and perceived support. Employing a structural equation modeling (SEM) framework, the study investigates: (i) the direct effects of depression, anxiety, and stress on PSMU; (ii) the mediating role of social media use duration; and (iii) the moderating role of perceived social support. By offering a theoretically grounded and empirically supported model, the findings aim to inform targeted strategies for prevention and intervention, providing valuable guidance to educators, mental health practitioners, and policymakers working to mitigate the risks of digital overuse in this uniquely vulnerable student population.

## 2 Methods

### 2.1 Study Procedure and Participants

A cross‐sectional study was carried out among the Bangladesh admission test‐taking students at Jahangirnagar University. Data were collected in February 2025. During this exam period, the research team approached the test‐taking students who resided in the university dormitory. Participation in the study was voluntary, and the research team provided them with detailed information about the study objectives and the questionnaire. A semi‐structured questionnaire was used to collect data using a non‐probability sampling technique to recruit the admission students. Initially, 1163 respondents completed the survey, but after eliminating the incomplete surveys, a total of 1139 respondents were considered for final analysis in this study.

### 2.2 Measures

#### 2.2.1 Sociodemographic Information

The questionnaire consisted of sociodemographic information related to gender (male vs. female), age (16–19 years, 20–22 years), religion (Muslim, Hindu, or other), location of permanent residence (urban vs. rural areas), family type (nuclear vs. joint), father's and mother's education level (primary or below, secondary, higher, and graduate or above), health status (good vs. poor) and monthly family income which categorized as less than 20,000 Bangladeshi Taka (BDT), 20,000 to 40,000 BDT, and more than 40,000 BDT.

#### 2.2.2 Admission Test‐Related Variables

Most of the universities in Bangladesh allow two times admission tests, whereas we collected the admission test‐related information, including test‐taking status (first‐time vs. repeat test takers), and secondary and higher secondary exam results within grade point average (GPA). In addition, the participants were asked about their exam preparation, including whether they had received any coaching or professional guidance, and also asked whether they were satisfied with their previous mock test result or not, their desired institution/department where they wanted to be admitted, and collected their monthly study expenditure costs during the admission.

#### 2.2.3 Patient Health Questionnaire

The Patient Health Questionnaire (PHQ‐4) scale was used for rapid screening of depressive disorder and generalized anxiety disorder (Kroenke et al. 2009). The PHQ‐4 comprised of PHQ‐2 and GAD‐2 subscale, whereas PHQ‐2 items were worded as: “Over the last 2 weeks, how often have you been bothered by: little interest or pleasure in doing things?” and “over the last 2 weeks, how often have you been bothered by: feeling down, depressed, or hopeless?”. The next GAD‐2 items are worded as: “Over the last 2 weeks, how often have you been bothered by: feeling nervous, anxious, or on edge?” and “over the last 2 weeks, how often have you been bothered by not being able to stop or control worrying?” Both scales followed a 4‐point Likert approach and responses were rated as 0 (not at all) to 3 (nearly every day). A total score can range between 0 and 6, with a score of ≥ 3 indicating the positive screening of depression and anxiety, respectively.

#### 2.2.4 Perceived Stress Scale

Self‐perceived stress was assessed using a four‐item perceived stress scale (PSS‐4) to measure students’ stress levels (Cohen et al. 1983). These items were recorded, including two positively worded items (e.g., “In the last month, how often have you felt that you were unable to control the important things in your life?”) and two negatively worded items (e.g., “In the last month, how often have you felt confident about your ability to handle your personal problems?”). Each item is rated on a 5‐point Likert scale (0 = never to 4 = very often) with the range of 0 to 16. A score of 6 or higher indicates a high stress level in the study (Malik et al. 2020). The reliability of the scale is 0.76.

#### 2.2.5 Bergen Social Media Addiction Scale

The Bergen Social Media Addiction Scale (BSMAS) was used to measure the risk of individuals' PSMU. The BSMAS scale, developed by Andreassen (Andreassen et al. 2016), consists of six items/indicators: (1) salience, (2) mood modification, (3) tolerance, (4) withdrawal, (5) conflict, and (6) relapse, adapted from Bergen Facebook addiction scale (Andreassen et al. 2012). Each item was rated on a 5‐point Likert scale, and responses were recorded from 1 (very rarely) to 5 (very often). For example, items include “used social media so much that it has had a negative impact on your studies/work.” Total possible scores ranged from 6 to 30, and a score of 19 or higher indicates a greater PSMU risk (Bányai et al. 2017). In the present study, Cronbach's alpha was 0.78.

#### 2.2.6 Multidimensional Scale of Perceived Social Support (MSPSS scale)

The multidimensional scale of perceived social support (MSPSS) (Zimet et al. 1988) was used to measure the perceived social support. This MSPSS consists of 12 items rated on a 7‐point Likert scale (1 = very strongly disagree to 7 = very strongly agree). The MSPSS was assessed by three sources of social support: family support (four items), friend support (four items), and significant support by other person's (four items). The scores from all items were summed to compute the total score. The higher mean score indicates higher levels of perceived social support. In the current study, MSPSS scores were categorized as follows: low support (mean score 1.0–2.9), moderate support (3.0–5.0), and high support (5.1–7.0). The reliability of the scale is 0.90.

### 2.3 Ethics Statement

All research activities were conducted in accordance with the ethical guidelines adhering to the Declaration of Helsinki (1975, revised 2013). Ethical approval was obtained from CHINTA Research Bangladesh Ethics Committee (Reference: CHINTA/2025/02(5)). Prior to participation, all individuals received detailed information about the study's purpose, procedures, potential risks, and their right to withdraw at any time. Written informed consent was obtained from each participant. The collected data were kept confidential and used solely for academic research purposes. No financial or material incentives were offered for participation.

### 2.4 Statistical Analysis

Microsoft Excel 2019 was used for data entry and cleaning, while IBM SPSS Statistics for Windows, Version 26.0 (IBM Corp., Armonk, NY, USA) was used for statistical analyses. Descriptive statistics (i.e., frequency, percentages, mean, and standard deviation) were used to summarize the sociodemographic, behavioral, and psychosocial characteristics of the participants. Inferential statistics include chi‐square tests to assess bivariate associations between independent variables and the outcome variable, PSMU. Logistic regression was conducted to identify the potential factors associated with PSMU, adjusting for all the variables. Results were reported as adjusted odds ratios (AORs), with corresponding 95% confidence intervals (CIs). Multicollinearity was measured using VIF (<5) and tolerance (>0.2). Model adequacy was assessed using the omnibus test of model coefficients, Hosmer–Lemeshow goodness‐of‐fit test, and the Nagelkerke *R* ^2^. A *p*‐value < 0.05 was considered statistically significant.

To examine the complex relationships between psychological factors (depression, anxiety, stress), social media use behavior, and PSMU, SEM was conducted using the lavaan package in R (version 4.4.1). SEM enabled the evaluation of direct, indirect (mediated), and moderated effects. Model fit was assessed using standard indices: Chi‐square test (χ^2^), comparative fit index (CFI), Tucker–Lewis index (TLI), root mean square error of approximation (RMSEA), and standardized root mean square residual (SRMR). Bootstrapping with 5000 resamples was employed to estimate the standard errors and confidence intervals of indirect effects. A good model fit was indicated by non‐significant χ^2^, CFI and TLI ≥ 0.95, RMSEA ≤ 0.06, and SRMR ≤ 0.08 (Hu and Bentler 1998).

## 3 Results

### 3.1 Characteristics of the Study Participants

The study involved 1139 students, with 50.6% being male and 49.4% female. Most of the students belonged to the age group of 16 to 19 years (60.5%), from nuclear families (76.8%), and lived in rural areas (56.7%). More than half of the participants' mothers (54%) had completed their secondary education, whereas 45% of the participants' fathers had completed graduation or above. Besides, 81% of participants self‐reported that their health status was good, 9% reported smoking, and only 3% reported drug use. Additionally, 17% had experienced fractures of body parts, and 16% reported chronic disease problems (e.g., heart disease, jaundice, or breathing problems). Regarding admission‐related information, 64% were first‐time test takers, almost 80% received professional assistance for test preparation, and 35% were satisfied with their mock test results. According to the specified scale cutoff, overall 21.2% of participants were categorized as PSMU. The prevalence of depression was 39%, while approximately 58% and 60.2% of participants reported experiencing anxiety and high stress, respectively. Furthermore, 55.6% reported high perceived social support, while only 6.4% received low support (Table 1).

**TABLE 1:** Association between the studied variables and PSMU.

Variables	Total (*n*, %)	PSMU (242; 21.2%)	Non‐PSMU (897; 78.8%)	χ^2^ test value	*p‐*value
**Socio‐demographic variables**					
**Gender**					
Male	576 (50.6%)	139 (24.1%)	437 (75.9%)	5.797	**0.016**
Female	563 (49.4%)	103 (18.3%)	460 (81.7%)		
**Age**					
16–19 years	689 (60.5%)	135 (19.6%)	554 (80.4%)	2.260	0.133
20–22 years	428 (37.6%)	100 (23.4%)	328 (76.6%)		
**Permanent residence**					
Rural	646 (56.7%)	127 (19.7%)	519 (80.3%)	2.115	0.146
Urban	491 (43.1%)	114(23.2%)	377 (76.8%)		
**Family type**					
Joint	256 (22.5%)	61 (23.8%)	195 (76.2%)	1.346	0.246
Nuclear	875 (76.8%)	179 (20.5%)	696 (79.5%)		
**Father's education**					
Primary or below	170 (15%)	36 (21.2%)	134 (78.8%)	1.936	0.380
Secondary	457 (40%)	88 (19.3%)	369 (80.7%)		
Graduate or above	506 (45%)	116 (22.9%)	390 (77.1%)		
**Mother's education**					
Primary or below	197 (17%)	40 (20.3%)	157 (79.7%)	0.682	0.711
Secondary	610 (54%)	126 (20.7%)	484 (79.3%)		
Graduate or above	325 (29%)	74 (22.8%)	251 (77.2%)		
**Monthly family income (BDT)**					
<20,000	224 (20%)	49 (21.9%)	175 (78.1%)	0.066	0.967
20,000–40,000	650 (57%)	137 (21.1%)	513 (78.9%)		
>40,000	265 (23%)	56 (21.1%)	209 (78.9%)		
**Self‐reported health status**					
Good	920 (81%)	188 (20.4%)	732 (79.6%)	2.143	0.143
Poor	212 (19%)	53 (25.0%)	159 (75%)		
**Cigarette/Tobacco smoking (1 year)**					
Yes	102 (9%)	40 (39.2%)	62 (60.8%)	21.448	**<0.001**
No	1,034 (91%)	202 (19.5%)	832 (80.5%)		
**Fracture of body parts (hand/feet/others)**					
Yes	196 (17%)	59 (30.1%)	137 (69.9%)	11.095	**<0.001**
No	943 (83%)	183 (19.4%)	760 (80.6%)		
**Chronic disease problems (heart/breathing/jaundice etc.)**					
Yes	183 (16%)	55 (30.1%)	128 (69.9%)	9.589	**0.002**
No	945 (83%)	187 (19.8%)	758 (80.2%)		
**Admission‐related variables**					
**Appearance in the admission test**					
First timer	730 (64%)	143 (19.6%)	587 (80.4%)	3.230	0.072
Second timer	406 (36%)	98 (24.1%)	308 (75.9%)		
**GPA in SSC**					
Poor	66 (6%)	17 (25.8%)	49 (74.2%)	0.921	0.631
Moderate	201 (18%)	41 (20.4%)	160 (79.6%)		
High	867 (76%)	182 (21.0%)	685 (79%)		
**GPA in HSC**					
Poor	163 (14%)	39 (23.9%)	124 (76.1%)	1.401	0.496
Moderate	360 (32%)	79 (21.9%)	281 (78.1%)		
High	611 (54%)	122 (20.0%)	489 (80.0%)		
**Coached by professional coaching centers**					
Yes	899 (79%)	185 (20.6%)	714 (79.4%)	1.276	0.259
No	238 (21%)	57 (23.9%)	181 (76.1%)		
**Satisfied with previous mock test performance**					
Yes	397 (35%)	58 (14.6%)	339 (85.4%)	16.044	**<0.001**
No	742 (65%)	184 (24.8%)	558 (75.2%)		
**Monthly expenditure during admission test (BDT)**					
<5000	297 (26%)	68 (22.9%)	229 (77.1%)	0.882	0.643
5000–10,000	580 (51%)	117 (20.2%)	463 (79.8%)		
>10,000	248 (22%)	53 (21.4%)	195 (78.6%)		
**Social media using related variables**					
**Social media using duration per day**					
<30 min	157 (13.8%)	7 (4.5%)	150 (95.5%)	181.79	**<0.001**
30–60 min	204 (17.9%)	16 (7.8%)	188 (92.2%)		
1–2 h	280 (24.6%)	42 (15%)	238 (85%)		
2–4 h	294 (25.8%)	68(23.1%)	226 (76.9%)		
>4 h	204 (17.9%)	109 (53.4%)	95 (46.6%)		
**Mental health and social support**					
**Depression**					
Yes	444 (39.0%)	126 (28.4%)	318 (71.6%)	22.118	**<0.001**
No	695 (61.0%)	116 (16.7%)	579 (83.3%)		
**Anxiety**					
Yes	661 (58.0%)	168 (25.4%)	493 (74.6%)	16.363	**<0.001**
No	478 (42.0%)	74 (15.5%)	404 (84.5%)		
**Self‐perceived stress**					
High stress	686 (60.2%)	182 (26.5%)	504 (73.5%)	28.781	**<0.001**
No stress	453 (39.8%)	60 (13.2%)	393 (86.8%)		
**Perceived social support**					
Low support	73 (6.4%)	26 (35.6%)	47 (64.4%)	25.657	**<0.001**
Moderate support	433 (38.0%)	114 (26.3%)	319 (73.7%)		
High support	633 (55.6%)	102 (16.1%)	531 (83.9%)		

### 3.2 Association Between the Studied Variables and PSMU

Table 1 illustrates the relationships between study variables and PSMU use among admission test takers in Bangladesh. Gender was significantly associated with PSMU, with male students (24.1%) showing higher rates than females (*χ* ^2^ = 5.797, *p* = 0.016). Smokers were more likely to have higher rates of PSMU (39.2%), and the association was statistically significant (χ^2^ = 21.448, *p* < 0.001). Students who had experienced fractures of body parts (30.1%) and those with chronic diseases (30.1%) had significantly higher rates of PSMU (χ^2^ = 11.095, *p* < 0.001 and χ^2^ = 9.589, *p* < 0.002, respectively). Participants dissatisfied with their previous mock test results were more likely to have higher rates of PSMU (χ^2^ = 16.044, *p* < 0.001). Importantly, those who used social media for more than 4 h daily had significantly higher addiction rates than those who used it for less than 30 min (χ^2^ = 181.79, *p* < 0.001). Those with mental health issues such as depression 28.4%; χ^2^ = 22.118, *p* <0.001), anxiety (χ^2^ = 16.363, *p* < 0.001), and self‐perceived stress (χ^2^ = 28.781, *p* < 0.001) were also significantly more likely to experience PSMU. Additionally, perceived social support was significantly associated with PSMU, with participants reporting low and moderate support showing higher PSMU levels compared to those with high support (χ^2^ = 25.657, *p* < 0.001).

### 3.3 Predictive Factors of PSMU Among Test‐Taking Students

The analysis in Table 2 highlights several significant risk factors for PSMU among admission test takers. Tobacco or cigarette smoking emerged as a key factor, with smokers having 1.89 times higher odds of developing PSMU compared to non‐smokers (95% CI = 1.115–3.208; *p* = 0.018). Similarly, students who had experienced fractures showed 1.70 times greater odds of addiction (95% CI = 1.101–2.595; *p* = 0.016), while no significant association was observed among those with chronic diseases. Psychological factors also played a significant role. Depression was associated with a 1.6‐fold increase in the odds of PSMU (OR = 1.60, 95% CI = 1.10–2.34; *p* = 0.014), while high stress levels increased the odds by 1.65 times (OR = 1.65, 95% CI = 1.03–2.64; *p* = 0.036). Individuals who used social media for less than 30 min per day had substantially lower odds of addiction (OR = 0.045, 95% CI = 0.018–0.110; *p* < 0.001). The odds slightly increased with longer usage: 30–60 min/day (OR = 0.08, 95% CI = 0.042–0.149; *p* < 0.001), 1–2 h/day (OR = 0.158, 95% CI = 0.09–0.26; *p* < 0.001), and 2–4 h/day (OR = 0.27, 95% CI = 0.18–0.42; *p* < 0.001). Individuals with moderate social support have 1.51 times higher odds of exhibiting PSMU compared to those with high social support (OR = 1.51, 95% CI: 1.052–2.167, *p* = 0.025).

**TABLE 2:** Predictors of social media addiction among university admission test‐taking students.

Variables	B	S.E	AOR	95% CI	*p*‐value
**Socio‐demographic variables**					
**Gender**					
Male	0.245	0.199	1.277	0.865, 1.886	0.219
Female	reference				
**Age**					
16–19 years	0.065	0.204	1.067	0.715, 1.593	0.750
20–22 years	reference				
**Permanent residence**					
Rural	−0.077	0.193	0.926	0.634, 1.353	0.691
Urban	reference				
**Family type**					
Joint	0.374	0.209	1.453	0.964, 2.191	0.074
Nuclear	reference				
**Father education**					
Primary or below	0.365	0.372	1.440	0.695, 2.985	0.326
Secondary	−0.109	0.226	0.897	0.576, 1.396	0.630
Graduate or above	reference				
**Mother education**					
Primary or below	−0.275	0.372	0.760	0.367, 1.574	0.459
Secondary	−0.094	0.233	0.911	0.577, 1.437	0.687
Graduate or above	reference				
**Monthly family income (BDT)**					
<20,000	0.228	0.309	1.256	0.685, 2.302	0.461
20,000–40,000	0.093	0.225	1.098	0.707, 1.705	0.678
>40,000	reference				
**Self‐reported health status**					
Good	0.302	0.229	1.353	0.863, 2.119	0.188
Poor	reference				
**Cigarette/Tobacco smoking (1 year)**					
Yes	0.637	0.270	1.891	1.115, 3.208	**0.018**
No	reference				
**Fracture of body parts (hand/feet/others)**					
Yes	0.525	0.219	1.691	1.101, 2.595	**0.016**
No	reference				
**Chronic disease problems (heart/breathing/jaundice, etc.)**					
Yes	0.054	0.226	1.056	0.678, 1.645	0.810
No	reference				
**Admission‐related variables**					
**Appearance in the admission test**					
First Timer	−0.053	0.212	0.949	0.626, 1.437	0.803
Second Timer	reference				
**GPA in SSC**					
Poor	0.025	0.378	1.026	0.489, 2.151	0.947
Moderate	−0.143	0.249	0.867	0.532, 1.413	0.567
High	reference				
**GPA in HSC**					
Poor	0.168	0.276	1.184	0.690, 2.031	0.541
Moderate	0.238	0.197	1.269	0.862, 1.869	0.227
High	reference				
**Coached by professional coaching centers**					
Yes	−0.061	0.227	0.940	0.603, 1.466	0.786
No	reference				
**Satisfied with previous mock test performance**					
Yes	reference				
No	0.207	0.203	1.230	0.826, 1.832	0.308
**Monthly expenditure during admission test (BDT)**					
<5000	0.238	0.260	1.269	0.762, 2.112	0.360
5000–10,000	−0.041	0.228	0.960	0.614, 1.500	0.857
>10,000	reference				
**Social media using related variables**					
**Social media using duration per day**					
<30 min	−3.100	0.458	0.045	0.018, 0.110	**<0.001**
30–60 min	−2.538	0.323	0.079	0.042, 0.149	**<0.001**
1–2 h	−1.847	0.248	0.158	0.097, 0.257	**<0.001**
2–4 h	−1.300	0.221	0.273	0.177, 0.420	**<0.001**
>4 h	reference				
**Mental health and social support**					
**Depression**					
Yes	0.475	0.193	1.608	1.101, 2.349	**0.014**
No	reference				
**Anxiety**					
Yes	0.270	0.231	1.311	0.834, 2.060	0.241
No	reference				
**Self‐perceived stress**					
High stress	0.501	0.239	1.651	1.034, 2.636	**0.036**
No stress	reference				
**Perceived social support**					
Low support	0.543	0.336	1.722	0.890, 3.330	0.106
Moderate support	0.412	0.184	1.510	1.052, 2.167	**0.025**
High support	reference				

### 3.4 Structural Equation Modeling of PSMU

**FIGURE 1:**
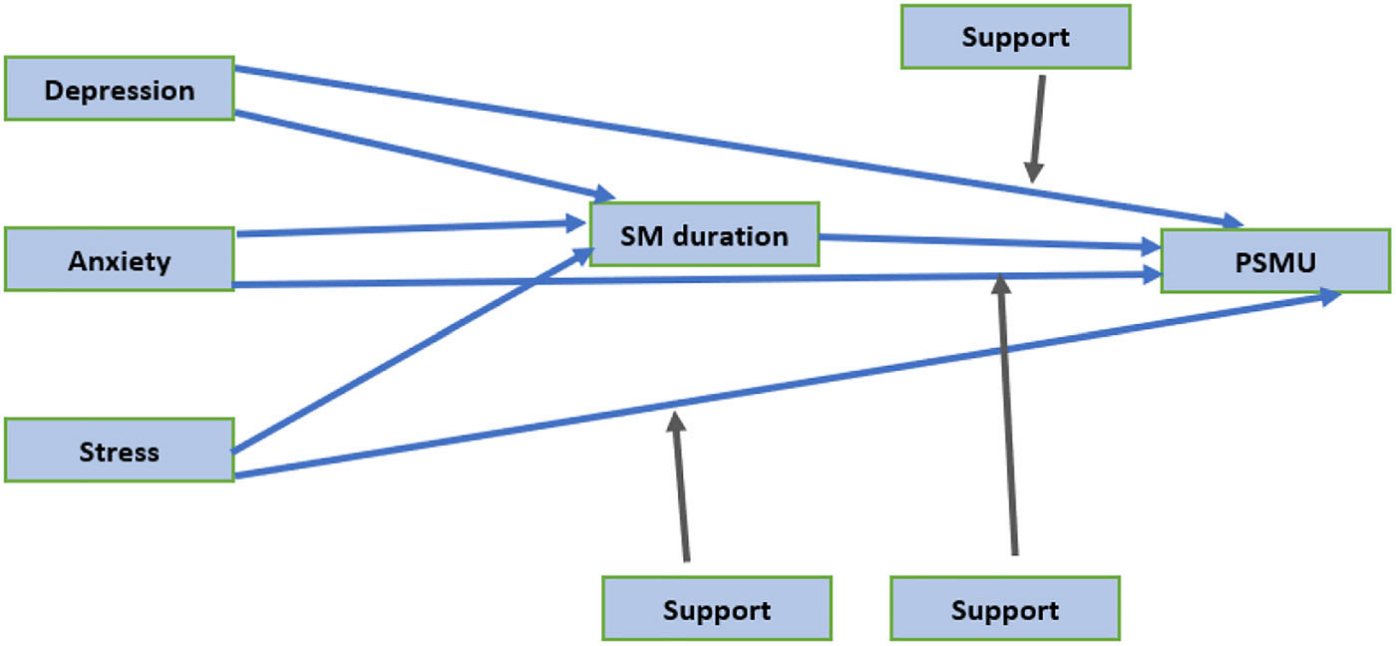
Structural equation modeling of PSMU.

A SEM approach was utilized to examine the direct, indirect (mediation), and moderated effects of depression, anxiety, and stress on PSMU, with social media (SM) use duration as a mediator and perceived social support as a moderator (Figure 1). The model demonstrated excellent fit to the data (*χ* ^2^(3) = 1.36, *p* = 0.715; CFI = 1.000; TLI = 1.019; RMSEA = 0.001 [90% CI: 0.001–0.036]; SRMR = 0.006), indicating that the hypothesized relationships were consistent with the observed data (Table 3).

**TABLE 3:** Structural equation modeling results of PSMU.

Effects	Estimate	SE	*p*‐value	Std. beta	CI lower	CI upper
**Direct effects on PSMU**						
Depression → PSMU	0.121	0.162	0.453	0.024	−0.214	0.427
Anxiety → PSMU	0.37	0.141	**0.009**	0.094	0.097	0.648
Stress → PSMU	0.271	0.056	**<0.001**	0.168	0.16	0.379
SM duration → PSMU	2.19	0.126	**<0.001**	0.447	1.939	2.439
**Mediation via social media use duration**						
Depression → SM duration → PSMU	−0.055	0.083	0.511	−0.011	−0.222	0.105
Anxiety → SM duration → PSMU	−0.033	0.074	0.651	−0.009	−0.182	0.115
Stress → SM duration → PSMU	0.089	0.03	**0.003**	0.055	0.028	0.149
**Total effects on PSMU**						
Depression → PSMU	0.066	0.192	0.729	0.013	−0.325	0.44
Anxiety → PSMU	0.337	0.16	**0.036**	0.086	0.031	0.655
Stress → PSMU	0.36	0.063	**<0.001**	0.223	0.234	0.481
**Moderation by perceived social support**						
Depression × support → PSMU	−0.005	0.203	0.98	−0.001	−0.394	0.404
Anxiety × support → PSMU	−0.218	0.227	0.338	−0.035	−0.664	0.219
Stress × support → PSMU	0.039	0.206	0.848	0.007	−0.346	0.462

#### 3.4.1 Direct Effects

Among the psychological predictors, anxiety (*β* = 0.37, *p* = 0.009, 95% CI [0.097, 0.648]) and stress (*β* = 0.27, *p* < 0.001, 95% CI [0.160, 0.379]) were both significantly and positively associated with PSMU. This suggests that higher levels of anxiety and stress are linked to greater problematic engagement with social media. In contrast, depression showed no significant direct association with PSMU (*β* = 0.12, *p* = 0.453). Additionally, social media use duration emerged as a strong predictor of PSMU (*β* = 2.19, *p* < 0.001), indicating that the amount of time spent on social media substantially contributes to problematic usage patterns (Table 3).

#### 3.4.2 Mediation via Social Media Use Duration

The mediation analysis revealed a significant indirect effect of stress on PSMU through SM duration (indirect *β* = 0.089, *p* = 0.003, 95% CI [0.028, 0.149]), suggesting that one pathway through which stress influences PSMU is by increasing the amount of time spent on social media. However, the indirect effects of depression (indirect *β* = –0.055, *p* = 0.511) and anxiety (indirect *β* = –0.033, *p* = 0.651) through SM duration were non‐significant. These findings indicate that while stress operates partially through behavioral engagement with social media, the same mechanism may not explain the effects of anxiety or depression (Table 3).

#### 3.4.3 Total Effects

The total effect of stress on PSMU remained significant even when the indirect pathway was included (total *β* = 0.36, *p* < 0.001), underscoring the robust influence of stress on problematic usage. The total effect of anxiety was also significant (*β* = 0.337, *p* = 0.036), while depression continued to exhibit a non‐significant total effect (*β* = 0.066, *p* = 0.729), further reinforcing the minimal role of depressive symptoms in driving PSMU in this sample (Table 3).

#### 3.4.4 Moderation by Perceived Social Support

Contrary to expectations, none of the interaction terms between perceived social support and psychological symptoms were significant. Specifically, social support did not moderate the relationships between PSMU and depression (*β* = –0.005, *p* = 0.980), anxiety (*β* = –0.218, *p* = 0.338), or stress (*β* = 0.039, *p* = 0.848). These results suggest that perceived social support does not buffer the effects of mental health symptoms on PSMU in this sample, highlighting that support alone may not be sufficient to mitigate the impact of internalizing symptoms on problematic online behavior (Table 3).

## 4 Discussion

This study sought to examine the socio‐demographic, health‐related, and psychosocial predictors of PSMU among university admission test‐taking students in Bangladesh—a population that has received minimal scholarly attention despite facing intense academic and emotional pressures. Our findings offer novel insights into the behavioral and psychological mechanisms underlying PSMU in this uniquely vulnerable cohort. The results revealed that male gender, cigarette or tobacco use, a history of physical fractures, and elevated levels of perceived stress were significant factors for PSMU. Additionally, students experiencing depressive symptoms and those reporting moderate perceived social support were more likely to engage in problematic social media use. The structural equation modeling further demonstrated that anxiety, stress, and social media use duration were significant predictors of PSMU, while stress also exerted an indirect effect through increased social media use duration. Contrary to expectations, perceived social support did not significantly moderate the relationship between psychological distress and PSMU. Collectively, these findings underscore the complex interplay between physical health, mental well‐being, and behavioral engagement with digital platforms, emphasizing the need for multifaceted prevention strategies tailored to this high‐risk student group.

The present study found that 21.2% of university admission test‐taking students in Bangladesh exhibited symptoms indicative of PSMU. This prevalence is notable given the relative lack of prior research on this specific population. Compared to other Bangladeshi studies, the observed rate appears moderately lower. For instance, Karim et al. (2023) reported a Facebook addiction prevalence of 29.4% among medical students (Karim et al. 2023), while Al‐Mamun et al. (2022) reported that 29.1% of university students were problematic Facebook users (Al‐Mamun et al. 2022). These higher prevalence rates among enrolled students may be attributed to more consistent access to internet‐enabled devices, greater social autonomy, and increased exposure to digital environments within the university setting.

In the global context, the observed prevalence aligns closely with findings from other countries. For instance, a study conducted in Vienna among college students reported that 22.7% of students were addicted to social media (Eichenberg et al. 2024), and Chen et al. (2020) also found a similar rate of 21.7% for Taiwanese students (Chen et al. 2020). These similar estimates suggest that the level of problematic use observed in our study is not unique to the Bangladeshi context and may reflect a broader pattern of emerging digital dependence during late adolescence and early adulthood. However, substantially higher rates were found in Saudi Arabia, where 55.2% of university students exhibited PSMU (Alfaya et al. 2023). Such variations may stem from differences in digital infrastructure, parental supervision, media literacy, and psychosocial support systems across countries.

These comparisons highlight the importance of contextualizing digital behavior within sociocultural and educational frameworks. While Bangladeshi admission test‐takers may exhibit lower rates of PSMU compared to their enrolled peers, this does not negate their vulnerability. The transitional nature of this phase, marked by intense academic stress, career uncertainty, and emotional isolation, can create fertile ground for maladaptive coping behaviors, including compulsive social media engagement (Anto et al. 2023; He and Zhu 2024). If left unaddressed, such behaviors may not only disrupt academic performance but also impair mental well‐being and long‐term educational outcomes. These findings highlight the critical need for early screening and the development of preventive strategies targeting students at this stage. Tailored interventions promoting digital literacy, stress management, and emotional resilience could serve to reduce the risk of PSMU and enhance students' capacity to succeed in their academic pursuits.

Our findings indicate that tobacco or cigarette smokers had a significantly 1.89 times higher risk of PSMU than nonsmokers. This result is consistent with a prior study in Bangladesh, which showed that smokers were more likely to engage in PSMU (Islam et al. 2021). Global evidence further supports this relationship. A systematic review and meta‐analysis found that exposure to tobacco‐related content on social media increases the likelihood of both smoking and digital media engagement (Donaldson et al. 2022). Although this study focused more on content exposure than behavior per se, it points to a potentially reciprocal relationship in which smokers not only consume but also engage with content that normalizes or promotes smoking, thereby reinforcing social media usage patterns (Donaldson et al. 2022). This loop may be further intensified by the type of engagement on social media. Cheng et al. (2023) distinguished between passive (e.g., browsing, scrolling) and active (e.g., posting, messaging) usage patterns, finding that passive engagement is more strongly associated with negative psychological outcomes, including depression and affective disturbances. Smokers may be more inclined toward such passive consumption seeking distraction or escape, which in turn can elevate the risk of PSMU (Cheng et al. 2023). Neurobiological mechanisms may also underlie this relationship. Ranker et al. (2024) found that smokers were more likely to develop behavioral addictions such as PSMU, possibly due to shared alterations in dopaminergic signaling and impulse control (Ranker et al. 2024). Both nicotine consumption and social media use activate the mesolimbic dopamine system, which plays a central role in reward processing and reinforcement learning. This has been highlighted by previous research reporting that excessive social media use shares neural correlates with substance use addiction (Tereshchenko 2023). In high‐pressure environments such as those experienced by Bangladeshi university admission test‐takers, this relationship may be amplified by academic stress, impulsivity, and cultural acceptance of both smoking and social media use. Addressing this dual burden requires integrated interventions, including stress management programs, mental health education, and algorithmic transparency campaigns, which could help disrupt the feedback loop between smoking and PSMU.

The present study identified social media use duration as a potential risk factor of PSMU. Our study demonstrated that students who used social media for less than 30 min per day had a significantly lower risk of developing PSMU compared to those who used it for more than 4 h daily. This finding is consistent with prior research (Pelin YILDIZ et al. 2023; Simsek et al. 2019). For instance, a recent study from Iraq reported that using social media for more than four hours daily significantly increased the risk of PSMU (Shanshal et al. 2024). Similarly, another study found a strong association between more than seven hours of daily use and social media addiction among Turkish students (Sümen and Evgin 2021). A Bangladeshi study reported that participants using social media for more than 5 h daily were more likely to experience Facebook addiction (Al‐Mamun et al. 2022).

Results from the structural equation modeling corroborated this relationship, demonstrating a strong and statistically significant direct effect of us duration on PSMU, indicating that increased screen time substantially elevates the risk of addictive behavior. These outcomes reinforce the notion that usage time is not merely a behavioral metric but a psychological indicator of vulnerability to maladaptive engagement (Kardefelt‐Winther 2014).

Several interconnected mechanisms can explain this association. Excessive social media use is associated with compulsive behaviors such as mindless scrolling, emotional reactivity, and instant gratification, all of which create a reward‐seeking behavioral loop (Kardefelt‐Winther 2014; Primack et al. 2017; Shanshal et al. 2024). Moreover, prolonged social media use is linked to reduced social interaction, disrupts sleep quality, and negatively impacts academic performance (Sümen and Evgin 2021; Shanshal et al. 2024). These adverse effects, particularly in an academically pressured group such as university test‐takers, may further exacerbate the cycle of digital dependence.

Importantly, the mediation analysis revealed that perceived stress exerted an indirect effect on PSMU through social media use duration, suggesting that individuals under high stress may increase their time on social media platforms as a coping mechanism. This pathway did not reach statistical significance for depression and anxiety, indicating that stress may uniquely influence PSMU via increased engagement time.

Parallel to usage duration, depression, anxiety, and perceived stress were also significantly associated with PSMU, in accordance with global evidence (Bányai et al. 2017; Woods and Scott 2016; Sümen and Evgin 2021). For instance, a study conducted among US adults reported that compulsive social media use was correlated with anxiety and depression (Primack et al. 2017), while a Scottish study found associations between PSMU and anxiety, depression, and low self‐esteem (Woods and Scott 2016). In Afghanistan, depressive symptoms were moderately to strongly associated with PSMU, confirming that individuals with depression are at heightened risk of excessive social media engagement (Haand and Shuwang 2020). Similarly, a study in Turkey showed a positive and significant relationship between perceived stress levels and PSMU (Sarialioğlu and Oluç 2024), and a study from China demonstrated that participants experiencing stress had a 1.19 times higher likelihood of PSMU, with depression emerging as another key contributing factor (Cui et al. 2023).

These results reflect the self‐medication hypothesis and the CIUT, which posits that individuals with poor mental health may use social media to alleviate negative emotions (Haand and Shuwang 2020; Kardefelt‐Winther 2014). Social media interactions, such as receiving likes, comments, or shares, provide brief mood enhancements via dopamine surges, mimicking pharmacological reward systems. However, this relief is temporary and often followed by emotional withdrawal or regret, creating a maladaptive feedback loop of prolonged engagement and worsened psychological symptoms. Furthermore, algorithmic targeting based on user behavior may amplify exposure to emotionally triggering or comparative content, especially for users already experiencing depressive symptoms, thereby reinforcing negative affect and driving longer usage sessions (Sarialioğlu and Oluç 2024). This dual mechanism, neurobiological reinforcement and digital platforms algorithms, can intensify PSMU in already vulnerable individuals. For Bangladeshi test‐takers, this cycle is compounded by intense academic pressure and limited access to mental health support. Therefore, integrated interventions are essential to address both digital behavior and psychological distress simultaneously.

Our study revealed that participants with moderate perceived social support had a 1.51‐fold higher risk of PSMU compared to those with high perceived social support. This finding is consistent with existing literature emphasizing the protective role of robust social support in mitigating the risk of problematic online behavior. For example, a previous study reported that while both real‐life and online social support correlated with PSMU, only real‐life social support effectively reduced depression, anxiety, and feelings of social isolation (Meshi and Ellithorpe 2021). Similarly, a Turkish study reported that individuals who received higher levels of support from family and friends exhibited significantly lower levels of social media addiction (Bilgin and Taş 2018). Conversely, our results suggest that moderate support, although generally beneficial, may not sufficiently buffer against the adverse effects of stress‐induced social media overuse, particularly in academically high‐pressure scenarios. This aligns with the stress‐buffering hypothesis, indicating that the efficacy of perceived social support depends on its adequacy in relation to the intensity of stressors experienced (Kelly et al. 2019). This suggests that moderate support, though protective in general, may lose its efficacy in high‐pressure contexts like those faced by Bangladeshi university entrance examinees.

In the context of our SEM findings, the hypothesized moderating role of perceived social support on the relationship between psychological distress and PSMU was not statistically significant. This indicates that while social support generally contributes to mental resilience, in this specific high‐stress scenario, its moderating potential appears limited. It underscores the necessity of targeted, contextually sensitive interventions that bolster higher levels of real‐life support and directly address coping strategies to effectively reduce the likelihood of problematic social media engagement in vulnerable student populations.

## 5 Practical Implications

The findings of this study have several important implications for addressing PSMU among university admission test‐taking students. Educational institutions and policymakers should implement structured digital literacy programs that emphasize responsible online behavior, teaching students how to manage their social media engagement effectively. Such programs should specifically highlight the importance of moderation in social media use and encourage the development of robust offline social connections and support systems.

Given the significant associations between PSMU and psychological distress identified in this study, integrating comprehensive mental health support within academic environments is essential. Proactive mental health interventions, such as screening and early identification programs, accessible counseling services, and stress management training, should be routinely offered to students preparing for university admission tests. These initiatives could effectively mitigate common mental health issues such as anxiety, depression, and perceived stress, subsequently reducing the likelihood of PSMU.

Additionally, public health campaigns targeting tobacco use could indirectly alleviate PSMU risks by addressing shared underlying behavioral patterns. Considering the observed association between smoking behaviors and heightened PSMU risk, multifaceted campaigns combining tobacco cessation initiatives with digital wellness education might be particularly beneficial.

Moreover, academic institutions should consider providing targeted academic support services to help students manage academic dissatisfaction and stress. Personalized tutoring, mentoring programs, and stress‐reduction workshops can help students cope better with academic pressures, thereby reducing their reliance on social media as a maladaptive coping mechanism.

Collectively, these integrated interventions encompassing digital literacy, mental health support, substance use reduction, and targeted academic assistance present a comprehensive approach to effectively addressing and mitigating PSMU among university admission test‐taking students in Bangladesh.

## 6 Strength and Limitations

This study's key strengths include its comprehensive assessment of PSMU within a large, gender‐balanced sample (*N* = 1139, 50.6% male) of Bangladeshi university admission test‐taking students. The targeted examination of this specific and relatively underexplored population is notable, as this group experiences unique academic pressures and psychosocial stressors. Additionally, the inclusion of diverse socio‐demographic, behavioral, and psychological variables provides a multidimensional understanding of the factors influencing PSMU. By focusing explicitly on students preparing for university entrance exams, the study generates novel and contextually relevant evidence that can inform culturally appropriate interventions aimed at reducing PSMU among Bangladeshi youth.

Despite these strengths, several limitations should be acknowledged. First, the cross‐sectional design precludes causal inference and limits conclusions regarding the temporal ordering of observed associations. Second, the reliance on self‐reported data introduces potential social desirability and recall biases, possibly affecting the accuracy and reliability of responses. Third, sampling from a single university may limit the generalizability of findings to broader populations, suggesting caution when extrapolating results beyond similar contexts.

To address these limitations, future research would benefit from employing longitudinal study designs capable of establishing causal and temporal relationships. Additionally, adopting mixed‐methods approaches, incorporating qualitative techniques (e.g., in‐depth interviews or focus groups), could enrich the understanding of nuanced psychological and behavioral dynamics underlying PSMU. Furthermore, future investigations should expand their scope by examining platform‐specific patterns of social media use (e.g., TikTok, Instagram, Facebook), exploring underlying neurobiological mechanisms (e.g., dopamine sensitivity), and assessing additional psychosocial constructs such as FOMO, loneliness, and social anxiety. These directions would yield deeper insights into PSMU, providing a more comprehensive understanding of its etiology and potential mitigation strategies.

## 7 Conclusions

This study highlights the moderate level of problematic social media use among university admission test‐taking students in Bangladesh and identifies several risk factors. By focusing exclusively on this underrepresented yet high‐stress group, the study addresses a critical gap in the literature and offers novel insights into the correlates of PSMU in the South Asian context. Key contributing factors, including lower perceived social support, dissatisfaction with academic outcomes, and the presence of mental and physical health challenges (e.g., recent illness or injury) provide a multidimensional view of the drivers of PSMU. These findings add nuance to the existing body of research and have practical implications for designing culturally sensitive, evidence‐based interventions aimed at improving digital well‐being and mental health among Bangladeshi youth.

Future research should adopt longitudinal designs and incorporate objective measures of social media use and mental health to strengthen causal inferences and enhance generalizability. Such efforts will be instrumental in guiding policy development, educational programming, and mental health support tailored to the unique needs of this vulnerable population.

## Author Contributions

Conceptualization: Md. Majharul Islam, Mohammed A. Mamun, and Firoj Al‐Mamun. Methodology: Md. Majharul Islam, Mohammed A. Mamun, and Firoj Al‐Mamun. Software: Md. Majharul Islam and Firoj Al‐Mamun. Data curation: Md. Majharul Islam, S. M. Rakibul Hasan, Firoj Al‐Mamun. Investigation: Md. Majharul Islam, Akihiro Masuyama, Farzana Tayeeba, Md. Faruk Islam, Nayeem Hasan Obhi, Miftahul Jannat Tahia, Ayesha Siddika Khan Sayma, Moneerah Mohammad Almerab, Debasruti Ghosh, Saurabh Raj, Firoj Al‐Mamun. Validation: S. M. Rakibul Hasan, Akihiro Masuyama, Farzana Tayeeba, Md. Faruk Islam, Nayeem Hasan Obhi, Miftahul Jannat Tahia, Ayesha Siddika Khan Sayma, Moneerah Mohammad Almerab, Debasruti Ghosh, Saurabh Raj, Mohammed A. Mamun, Firoj Al‐Mamun. Formal analysis: Md. Majharul Islam, S. M. Rakibul Hasan, Firoj Al‐Mamun. Supervision: Mohammed A. Mamun, Firoj Al‐Mamun. Funding acquisition: Moneerah Mohammad Almerab. Visualization: S. M. Rakibul Hasan, Akihiro Masuyama, Farzana Tayeeba, Md. Faruk Islam, Nayeem Hasan Obhi, Miftahul Jannat Tahia, Ayesha Siddika Khan Sayma, Moneerah Mohammad Almerab, Debasruti Ghosh, Saurabh Raj, Mohammed A. Mamun, Firoj Al‐Mamun. Project administration: Md. Majharul Islam, Farzana Tayeeba, Md. Faruk Islam, Nayeem Hasan Obhi, Miftahul Jannat Tahia, Ayesha Siddika Khan Sayma, Moneerah Mohammad Almerab, Mohammed A. Mamun, Firoj Al‐Mamun. Resources: Farzana Tayeeba, Md. Faruk Islam, Nayeem Hasan Obhi, Miftahul Jannat Tahia, Ayesha Siddika Khan Sayma, Moneerah Mohammad Almerab. Writing – original draft: Md. Majharul Islam, S. M. Rakibul Hasan, Akihiro Masuyama, Firoj Al‐Mamun. Writing – review and editing: Md. Majharul Islam, S. M. Rakibul Hasan, Akihiro Masuyama, Farzana Tayeeba, Nayeem Hasan Obhi, Miftahul Jannat Tahia, Ayesha Siddika Khan Sayma, Moneerah Mohammad Almerab, Debasruti Ghosh, Saurabh Raj, Mohammed A. Mamun, Firoj Al‐Mamun.

## Conflicts of Interest

The authors declare no conflicts of interest.

## Peer Review

The peer review history for this article is available at https://publons.com/publon/10.1002/brb3.70911.

## Data Availability

Data is available from the corresponding author upon reasonable request.
